# Validity of the Nurses’ health study physical activity questionnaire in estimating physical activity in adults with rheumatoid arthritis

**DOI:** 10.1186/s12891-017-1589-y

**Published:** 2017-05-31

**Authors:** Thomas Quinn, Michelle Frits BS, Johan von Heideken, Christine Iannaccone, Nancy A. Shadick, Michael Weinblatt, Maura D. Iversen

**Affiliations:** 1 University of Massachusetts Medical School, Worcester, MA USA; 2 Division of Rheumatology, Immunology & Allergy, Brigham & Women’s Hospital, Boston, MA USA; 3 Department of Women and Children’s Health Karolinska Institutet, Stockholm, Sweden; 4 Harvard Medical School, Boston, MA USA; 5 Department of Physical Therapy, Movement and Rehabilitation Sciences, Bouvé College of Health Sciences, Northeastern University, 360 Huntington Avenue Rm 301c Robinson Hall, Boston, MA 02115 USA

**Keywords:** Physical activity, Rheumatoid arthritis, Measurement validity

## Abstract

**Background:**

Patients with rheumatoid arthritis (RA) demonstrate reduced aerobic capacity, excess cardiovascular risk, mobility limitations and are less physically active than their healthy peers. Physical activity may decrease RA disease activity through its anti-inflammatory effects and psychological and health benefits. To successfully manage RA symptoms and reduce cardiovascular risks associated with RA through increased physical activity (PA), accurate physical activity assessments are critical. Accelerometry is an objective physical activity measure, but not widely used. Validity of the Nurses’ Health Study physical activity questionnaire II (NHSPAQ) has not been determined for estimation of physical activity in RA. This study examined NHSPAQ validity in adults with RA compared to accelerometry-based metabolic equivalents determined (METs) and results of performance tests. We hypothesized NHSPAQ scores would correlate moderately (0.4–0.5) with accelerometer physical activity estimates.

**Methods:**

Thirty-five adults with RA (mean age [SD] 62 (Williams et. al, *Health Qual Life Outcomes* **10**:28, 2012) years, 28 females (80%) recruited from a hospital-based clinic registry participated in a one-week accelerometry trial. Medical data was compiled. Participants completed the NHSPAQ, a self-paced 20-m walk test, and modified timed step test. Participants wore an accelerometer for 7 consecutive days, then completed a physical activity log and another NHSPAQ. Metabolic equivalents (METs) were derived from NHSPAQ and accelerometers using standardized formulas. NHSPAQ METs were correlated with accelerometer METs and data from performance measures.

**Results:**

Average disease duration was 21 years (SD = 11), 63% patients took biologics. The average weekly METs reported were 29 (SD = 33) and accelerometer METs were 33 (SD = 22). NHSPAQ METs correlated moderately with accelerometer-derived METs (*r* = 0.48 95% CI (0.15–0.70). Self-reported PA correlated moderately with Step Test performance (*r* = 0.50 95% CI (0.18–0.72).

**Conclusion:**

Patients with RA exhibit low physical activity levels. General fitness measures were moderately correlated with physical activity levels. A moderate significant correlation existed between NHSPAQ and accelerometry METs. These preliminary data suggest the NHSPAQ may be useful to describe physical activity levels in this population.

## Background

Rheumatoid arthritis (RA) associated symptoms such as joint damage, joint pain, inflammation, and cardiovascular disease place these adults at greater risk of physical inactivity [1]. Even among those with well-controlled disease, adults with RA are less physically active than people who are disease-free [2, 3]. To successfully manage RA symptoms and reduce cardiovascular risks associated with RA through increased physical activity (PA) [4], an accurate measure of daily activity is essential for both the clinician and the patient.

Accelerometers, devices that measure bouts of PA above resting metabolic rate [5] are used in epidemiologic studies to ascertain estimates of PA [6]. From these measurements, metabolic equivalents of energy expenditure (METs) of tasks can be derived based on the duration and type of activity [7]. Maximal exercise testing enables the ascertainment of METs but requires expensive equipment and skilled clinicians whereas accelerometry provides an indirect estimate of METs and does not require large equipment (oxygen uptake equipment) nor skilled clinicians. One MET is operationally defined as the quantity of oxygen consumed while sitting at rest and equals 3.5 milliliters of oxygen per kilogram of body weight per minute. This MET notion represents a practical and understandable method for expressing the energy cost of physical activities in relation to resting metabolic rate [7]. Unfortunately, widespread clinical use of the maximal exercise testing to derive METs is not practical due to time constraints and costs. A simpler, more cost-effective method to accurately determine daily PA including estimated METs in adults with RA is clinically necessary to effectively manage RA symptoms through exercise and increased daily activity.

Physical activity is commonly defined, “as any bodily movement produced by skeletal muscles that requires energy expenditure” [8]. Physical activity questionnaires (PAQ) are often used in settings where more objective PA measurements are not practical or possible [9, 10]. Typically, PAQs gather information about leisure activity, exercise duration and intensity, and other measures of daily activity. Numerous PAQs exist and have been validated for use in specific cohorts where estimation of PA is particularly relevant [9, 11–13]. The International Physical Activity Questionnaire [11], Behavioral Risk Factor Surveillance System [12], Physical Activity Recall [13] and the Nurses’ Health Study Physical Activity Questionnaire II (NHSPAQ) [14, 15] are designed to describe PA behaviors, whereas the Neighborhood Environment Walkability Scale [16] is designed to describe aspects of the environment that promote PA or create barriers to PA. Each of these instruments has strengths and weaknesses. However, for a specific questionnaire to be used for a given population, it must first be validated in the target population. Initial validation studies of PAQs, such as the NHSPAQ, compared subject responses to items on the survey with PA diaries and logs [14]. The most accurate way to determine the validity of self-reported physical activity is through direct comparison with an objective measure.

The NHSPAQ is a self-reported PAQ originally developed for the Nurses’ Health Study [14, 15]. The questionnaire contains a series of items regarding PA such as modes of exercise, walking pace, flights of stairs climbed as well as items ascertaining sedentary activities (e.g. sitting). Responses include options related to the duration of activity for each mode, number of flights climbed and time spent sitting. The NHSPAQ was developed to report PA modes as well as derived METs. The calculation to derive METs uses the frequency, duration, and mode of PA (e.g. strength training), assuming moderate intensity for each activity and estimates of METs from the American College of Sports Medicine [17], summed across all modes reported [14].

We chose the NHSPAQ because it is a brief PAQ that has been validated for use in numerous large epidemiologic cohort studies of patients with chronic health conditions to ascertain METs of PA and examine the relationship between PA and disease outcomes [17–19]. However, the NHSPAQ has not been validated for use in patients with RA. Given the ever-increasing role of PA in the management of RA and to follow PA trends in large epidemiologic studies in these patients, it is pertinent to consider the NHSPAQ for clinical use in RA. While the gold standard comparison for assessing METs is indirect calirometry, we aimed to examine the concurrent validity of the NHSPAQ-derived METs and accelerometer-derived METs for determining PA levels in adults with RA. We hypothesized that NHSPAQ-derived METs would correlate moderately and positively with METs measured using accelerometers (r = 0.4 to 0.5). We further hypothesized that performance on the aerobic test would have a low to moderate correlation with self-reported PA levels (*r* = 0.3 to 0.5) given these are assessed at a single point in time, versus report of weekly PA.

## Methods

Institutional Human Subjects approval was obtained for this secondary study and all subjects consented to participate.

### Design and recruitment

This seven-day validation study took place from March, 2011 through September, 2012. Subjects were recruited through a clinical registry located in a large tertiary medical center. The RA registry [20] is a comprehensive database of over 1,000 patients with RA [21]. Patients in the registry met the following criteria: age 18 years or greater, doctor diagnosed RA or met the American College of Rheumatology criteria for RA [22] and agreed to participate. A statistician used a computer program to randomly select individuals from the registry who reported PA levels in the top decile and bottom decile of METs based on their responses to the NHSPAQ items. We selected subjects who were in the extreme ends of the PA spectrum in this sample to determine whether the questionnaire is useful and valid for individuals who are sedentary and highly active. An invitation letter was then mailed to these individuals explaining the study purpose and requesting participation. The research assistant contacted interested subjects by phone to screen subjects for eligibility. Eligibility included: no current engagement in swimming or other water-based exercise and medical history precluding participation (uncontrolled heart disease). Appointments were scheduled to coincide with an upcoming rheumatology appointment at the hospital’s rheumatology clinic.

### Protocol and measures

Demographic data, RA disease activity (biomarkers and physical examination data) and health status information (mood state and disability) were obtained from the RA registry for the most recent annual visit. During the visit, patients provided a blood sample for an array of clinical measures including: C-reactive protein (CRP), rheumatoid factor (RF), and anti-cyclic citrullinated peptide (anti-CCP) antibodies. Demographic data included sex, age, education, race, marital status, employment, and medications were obtained from the registry. RA disease activity was assessed using the Disease-Activity Score with C-Reactive Protein (DAS-CRP3) [23, 24]. The DAS-CRP3 combines a biomarker of disease activity − C-reactive protein − with an overall measure of disease activity including swollen and tender joints, as assessed by the rheumatologist and the patient’s global assessment of disease activity [23, 24]. DAS-CRP3 scores were obtained during the patient’s annual rheumatology visit and range from 0 to 10. Scores were classified as: low active (<3.2), moderately active (3.2–5.1) and highly active (>5.1). To ensure the most precise correlation of disease activity and physical activity possible, DAS-CRP3 values were taken from the annual visit closest to the patient intake date, an average of 4 months.

Mood state was assessed using the Mental Health Status Index (MHI-5) [25], a 5-item scale that assesses mental health status with scores range from 0 to 100, where higher scores indicate better mental health. This instrument has been validated and recommended as a tool to screen for mood disorders [26]. RA-related disability was assessed with the Multidimensional Health Assessment Questionnaire (MDHAQ), a valid and reliable measure of disability and function [27, 28] in adults with RA with scores ranging from 0 to 3 (worse function). The MDHAQ is a self-report questionnaire designed specifically for patients with RA and includes items regarding activities of daily living, pain, psychological status and global health status. All of the patient reported outcome measures are routinely used in studies of adults with RA.

At intake, participants completed an initial NHSPAQ [14] and performance tests conducted in random order, under the supervision of an experienced physical therapist who was blinded to subjects' PA status. The NHSPAQ contains items regarding the time and energy spent during the previous week performing various tasks such as climbing stairs, walking, running, and various forms of exercise. Additionally, the questionnaire gathers information about average gait speed and the amount of time spent in sedentary activities. The data are combined in a standardized algorithm to determine the METs expended during the previous week.

The Timed 20-meter Walk Test [29] was administered to provide information about average gait speed. This self-paced walk test is both valid and reliable in measuring average gait speed and functional performance in adults with arthritis [29]. A 20-meter course was measured and marked clearly at the starting and ending points. Patients were instructed to complete the course at their normal walking pace, making sure to continue through the end point without reducing their speed. To minimize outside influences on gait speed, the investigator remained stationary at the end of the course while timing each participant. The time taken to complete the course was recorded along with the number of steps taken. Each subject completed three trials of the course, and the average values were used in analysis.

Participants also completed a modified step test [30, 31] as a measure of resting, active, and recovery heart rates, and overall physical fitness. The step test provides valid and reliable estimates of aerobic capacity and respiratory exchange rate and consisted of three bouts of 3-min intervals on an 18 cm step while heart rate was monitored using a pulse oximeter. Heart rate was assessed at baseline, 5 s after completion of bout, and one minute after completion. Patients rested for one minute between each bout of exercise. Metabolic rate was determined from metabolic equivalents (METs) provided by data from the American College of Sports Medicine (ACSM) [32] for various stepping rates at specified step heights [31].

Upon completion of the two performance measures, participants were given an Actigraph© tri-axial accelerometer. Gyroscopic accelerometers quantitatively measure daily activity by recording peaks in acceleration in all three planes of movement (frontal, sagittal and transverse) over time. Studies have established the validity and reliability of accelerometers in many populations including adults with RA [33, 34]. From these measurements, METs can be calculated using subject weight and activity duration. Prior to administration, the device was synchronized with the subject’s height, weight, and date of birth. Participants were instructed to wear the device, centered over the hip on their dominant side. The investigator placed the device on the patient to ensure appropriate positioning of the accelerometer. Patients were also told not to participate in any water sport activities during the study as the device cannot be worn in the water. They were also instructed to remove the device while bathing, and could remove it before going to bed at night. Participants received written and oral instructions reviewing the protocols along with a daily activity log to record general patterns of activity throughout the day. Participants recorded the time the device was worn and removed each day, as well as any periods of rest that could be misinterpreted as periods of non-wear. Subjects wore the accelerometer for seven days. At the completion of the 7^th^ day, the device automatically stopped recording. Patients were instructed to complete a second NHSPAQ form at the end of the week and return the device, exercise log and NHSPAQ via mail using a pre-paid mailing carton (delivery information included).

### Analysis

All data was doubled entered and verified for accuracy by a third person. SAS Software, version (9.2) of the SAS System for [PC] © SAS Institute, was used to analyze demographic data and determine the correlation between the various performance measures and PA data. Standard METs for each activity reported via the NHSPAQ were calculated using the average duration, frequency, and intensity of the activity over the week. Individual METs from each activity were then summed and the total METs for the week were calculated. From the total weekly METs, average daily METs can be quickly and easily determined. METs for the timed-step test were calculated using values provided by the ACSM guidelines for an 18-cm step with step rates of 20–30 steps per minute. To calculate METs when stepping rates were outside this range, a linear regression was used to determine METs for all stepping rates. Stepping rate was plotted against the associated METs provided by the ACSM [32], and a formula relating the two variables was generated with R^2^-value 0.997.

Data from the accelerometry trials were analyzed using Actigraph software ©, which calculates total weekly METs by relating the duration and intensity of daily activities to the weight of the subject using a standardized formula. METs are reported in terms of the rate of oxygen consumption (in liters per minute) per kilogram of body weight, per minute of exercise. Correlations and corresponding 95% Confidence Intervals (CI) were used to determine associations with METs derived from accelerometry, performances tests and the NHSPAQ. Although subjects were asked not to engage in swimming during this study, five individuals reported swimming during the study period and removed the accelerometer when they swam. As a result, we examined total NHSPAQ METs with and without the METs associated with swimming in the total weekly METs calculation. A Bland-Altman plot [35] was used to analyze the agreement between NHSPAQ METs and those derived from the accelerometer. A Wilcoxon Ranked Sum Test examined whether accelerometer-derived METs differed between those individuals reporting high and low levels of PA engagement. Gpower software [36] was used to calculate power. With an alpha set at 0.05 and a correlation of 0.60, the study sample of 35 was sufficiently powered at 90%.

## Results

### Subjects

**Fig. 1:**
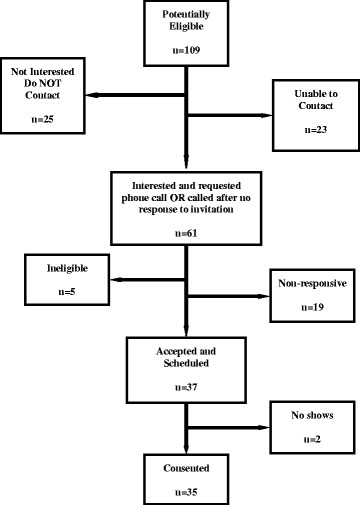
Recruitment Results for Accelerometry Trial in Rheumatoid Arthritis

One hundred and nine subjects were identified by the statistician and mailed information about the study. Sixty-one patients expressed interest in learning more about the study and gave permission to be called. Thirty-five patients consented to participate (Fig. 1). Of these 35, 34 (97%) were Caucasian, 28 (80%) were female, the mean age was 62 years (SD = 10), all were well educated (60% graduated college or had attended graduate school and had graduate degree) and most had longstanding RA. Patients exhibited moderate disease activity and good mental health status. For details see Table 1.

**Table 1:** Demographic and clinical characteristics of adults with rheumatoid arthritis participating in the accelerometry trial (*n* = 35)

Variable	Number (%)	Mean (SD)	Median (IQR)
Female	28 (80)		
Caucasian	34 (97)		
Age (y)		62 (10)	62 (52–68)
Married	30 (86)		
Employed	13 (37)		
Graduated high school/attended college	14 (40)		
Graduated college/attended graduate school	21 (60)		
Disease Duration (y)		21 (11)	19 (11–32)
Medications			
Biological DMARDs	22 (63)		
Methotrexate	18 (51)		
TNF inhibitor	18 (51)		
NSAIDs	12 (34)		
Steroids	14 (40)		
Disease markers			
Rheumatoid factor >15	21 (60)		
Anti-CCP positive	24 (69)		
Seropositive	25 (71)		
DAS-CRP3^a^		3.2 (1.5)	2.9 (1.9–4.3)
Disease activity (RADAI)		2.9 (2.1)	2.3 (1.2–4.7)
Self-reported outcomes (PROs)			
Multi-dimensional health Assessment Questionnaire (MDHAQ)		0.62 (0.54)	0.55 (0.1–1.0)
Quality of life (Euroqol Index)		0.8 (0.2)	0.8 (0.7–0.8)
Mental health score (MHI-5)^b^		79 (17.4)	83 (73–90)
Performance Measures			
20 m Timed walk (s)			
Number of steps		28 (4.5)	26 (24.3–30.7)
Time taken (seconds)		16.1 (3.8)	15.7 (12.9–17.4)
Timed Step test (METs)^a^		4.7 (0.8)	4.8 (4.3–5.1)

^a^Frequency Missing = 1

^b^Frequency Missing = 4

*SD* Standard deviation

*IQR* Interquartile range

Subjects averaged a metabolic rate of nearly 5 METs during the self-paced, modified step test. There was a low but significant correlation between performance on the modified step test and gait speed [Timed Walk (*r* = 0.39; 95% CI 0.05–0.64)]. Average baseline NHSPAQ-derived METs per week were 29 (SD = 33) and average NHSPAQ-derived METs on follow up were 25 (SD = 27) including those who reported swimming and 21 METS (SD = 21), excluding those who reported swimming. Average accelerometer-derived weekly METs measured slightly higher, at 33 METs (SD = 22). Most individuals reported walking or climbing stairs during the week as their primary modes of physical activity (Table 2).

**Table 2:** Average metabolic equivalents derived from NHSPAQ at baseline intake, and from accelerometers along with participation rates in specific physical activities among adults with RA (*n* = 35)

Measure	Calculated METs Mean (SD)	Median (IQR)
NHSPAQ II (Baseline)	29 (33)	21 (8–35)
NHSPAQ II (Week 1)	25 (27)	20 (6–30)
ActiGraph accelerometer (week 1)	33 (22)	28 (18–39)
Participation in various physical activity modes	Number (%)	
Walk	33 (94.3)	
Jog	5 (14.3)	
Run	1 (2.9)	
Swim	5 (14.3)	
Bicycle	12 (34.3)	
Calisthenics	10 (28.6)	
Tennis	1 (2.9)	
Stair climbing	32 (91.4)	

*SD* Standard deviation

*IQR*, Interquartile range

Baseline NHSPAQ-derived METs correlated moderately and significantly with the Modified Step Test (*r* =0.50; 95% CI = (0.18–0.72) but not with average gait speed (*r* = 0.06; 95% CI = −0.29–0.39). With respect to RA disease status, there was a moderate negative correlation between disease activity and NHSPAQ METs (*r* = 0.48; *p* = 0.007). Disability, as measured by the MDHAQ, did not correlate with either the NHSPAQ METS or the accelerometer-derived METS (Table 3).

**Table 3:** Correlations between metabolic equivalents (METs) derived from performance tests on day of intake, self-reported disability and from accelerometers among adults with RA (*n* = 35)

Variables	Correlation Strength	95% Confidence Limits
NHSPAQ METs		
With accelerometer METs	0.48	0.15–0.70
With modified step-test METs	0.50	0.18–0.72
With disability (MDHAQ)	−0.24	−0.54–0.12
Actigraph accelerometer METs		
With modified step-test METs	0.39	0.05–0.64
With walk-test speed	−0.50	−0.71–-0.19
With disability (MDHAQ)	−0.21	−0.51–0.15

**Fig. 2:**
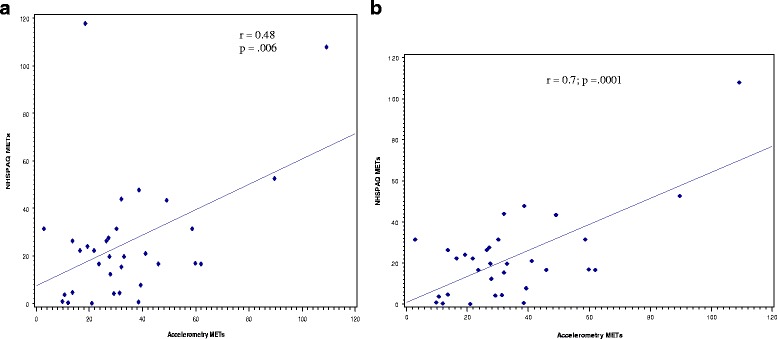
Correlation between metabolic equivalents (METs) derived from the NHSPAQ and from the accelerometer in adults with Rheumatoid Arthritis. **a** Scatterplot of Accelerometer METs with NHSPAQ Mets with Outlier included. **b** Scatterplot of Accelerometer METs with NHSPAQ Mets with Outlier excluded

A visual display of the relationship between METs-derived from the NHSPAQ and the accelerometer-derived METs in a scatterplot illustrates one outlier who reported more than 11 h of swimming during the week. This was confirmed by examining the patient’s weekly PA logs. As this accelerometer cannot be worn in water, excluding the subject from the analysis resulted in a stronger correlation between the NHSPAQ METs and the accelerometer METs (*r* = 0.7; 95% CI = 0.49–0.85). Thus, we plotted the relationship between NHSPAQ-derived METs and accelerometer-derived METs with this person removed and found a moderate and significant correlation between these two variables (*r* = 0.48; 95% CI = (0.15–0.70)) (Fig. 2).

**Fig. 3:**
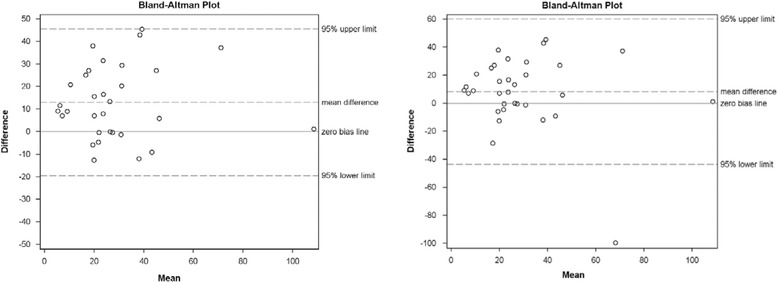
Bland-Altman plot illustrating relationship between NHSPAQ derived metabolic equivalents (METs) and METs derived from the accelerometer among adults with RA (*n* = 34). The figure on the left shows the Bland-Altman plot without the outlier and the one on the right with the outlier (NOTE: scale changes with deletion of outlier). The Bland-Altman plot indicates where the greatest variance in METs calculation occurs between the NHSPAQ and accelerometer trials. Upper and lower limits are +/- 1.96 standard deviations of the average difference between METs calculations

A Bland-Altman Plot was used to further examine the relationship between accelerometer-derived METs and NHSPAQ-derived METs. The plot indicates limits of agreement between the different two measures of PA, providing upper and lower limits that are +/− 1.96 standard deviations of the average difference between METs calculations. The magnitude of the agreement of these two measures is clinically insignificant, suggesting these two measures may be used interchangeably. (Fig. 3).

To further validate patient self-report of PA using the NHSPAQ, we examined accelerometer-derived METs between participants who self-reported high levels of PA and those self-reported low levels of PA, defined as being in the top and bottom decile of NHSPAQ METs. Data indicated that median accelerometer METs differed significantly between the two groups (median _high_ = 39; median _low_ = 24; z = 0.01; *p* = 0.007).

## Discussion

Given the evidence suggesting individuals with RA are at high risk for cardiovascular disease, this study aimed to examine the validity of the NHSPAQ in the estimation of daily PA levels in adults with RA, in order to determine whether the NHSPAQ might be useful in clinical practice or in large epidemiologic studies of PA in RA. Our data demonstrated a significant moderate correlation between NHSPAQ-derived METs and accelerometer-derived METs. While the correlation is modest, it is higher than correlations found between the NHSPAQ and PA diaries [14] and slightly higher than comparisons with other PA scales when used in adults with RA [37]. In a recent study of 50 adults with RA [38], the relationship between accelerometry and scores derived from the International Physical Activity Questionnaire (IPAQ) data was low to moderate [quadratic weighed kappa index 0.27 (0.06–0.48), *p* = 0.02]. Thus, our data suggest the NHSPAQ shows promise as a tool for determining PA levels in adults with RA. The Bland-Altman plot provides further confirmation that the NHSPAQ may provide a reasonable estimate of PA levels. However, the Bland Altman plot also suggests that among adults with RA who are sedentary, the relationship between NHSPAQ and accelerometer may be less reliable. Further, research is warranted to determine the effects of extreme activity levels (high or low) on the accuracy of the questionnaire.

In this study of patients with well established RA, PA levels were low. However, these PA levels are similar to data reported in other studies of PA in adults with RA [37–40]. In addition, we found a non-significant correlation between PA and physical function. While this result may be surprising, Conigliano et al. [38] also reported PA was not associated with function, as measured by the HAQ. It is difficult to draw any conclusion about the relationship of disease activity to PA as disease activity, measured by the DAS-CRP3, was assessed at annual visits, and in some cases this was an average of 4 months from the accelerometry trial intake.

There were low correlations between physical performance measures of aerobic status and gait speed with self-reported PA levels (NHSPAQ). Specifically, a moderate positive correlation was noted between NHSPAQ METs and performance on the modified step-test (*r* = 0.50; *p* = 0.003), indicating that individuals who are more physically active tended to perform at a higher level during this test. This finding is interesting clinically because the data suggest that the NHSPAQ, although used as an estimation of PA over one-week, might also be useful as an indicator of general fitness.

Overall, METs obtained from both the NHSPAQ and the accelerometers illustrate that persons with RA engage in far less PA than is recommended for the average person in the general population. The amount of time engaged in moderate to vigorous activity is shown to decrease dramatically across the population after the age of 16 [4], so it is to be expected that older persons would present as less physically active than their younger counterparts. However, when adjusting for age, patients with RA still vastly underperform in terms of their daily PA levels. The 2008 Physical Activity Guidelines for Americans recommends adults perform 500 to 1,000 METs of activity per week, which corresponds to at least 150 min of moderate exercise or 90 min of vigorous physical activity per week [41]. Updated guidelines for older adults recommends 150 min of moderate or 75 min of vigorous physical activity per week [42]. Inactivity is defined as less than 40 METs per week. According to these guidelines, the majority of participants in this study would be considered sedentary. This finding is supported by a 2012 study by Dr. Lee et al. [40], investigating PA in 176 adults with RA, 40% of the patients with RA were considered physically inactive by today’s guidelines with only 12% meeting even the minimum weekly exercise recommendations. In light of these and other similar findings, it is not surprising that the overall activity levels for patients with RA in this study were significantly lower than those indicated by ACSM. Clearly, although the benefits of exercise are well known, there are major obstacles that need to be overcome in order to increase PA levels in individuals with RA.

### Strengths/limitations

A major strength of this study is the use of the RA Registry that allowed us to ascertain biomarkers of disease activity without the need of additional time or resources and facilitated subject recruitment. We sampled patients based on prior self-report of PA to gather subjects at extreme end of PA levels and the investigator was blinded to PA levels at intake. Additionally, we compared a self-report PA questionnaire which includes activities that involve primary joints affected by RA and compared the data to simple clinical performance measures and an accelerometer. Each of these calculations rely on METs derived from standard formulas using ACSM criteria and do not require extensive equipment and costs associated with more comprehensive clinical tests. Limitations of the study include the small study sample. However, power calculations suggest we were sufficiently powered. Another limitation is the relative homogeneity of subjects with respect to race, socio-economic status, and high educational attainment. This may limit generalizability, as these patients may be more likely to accurately report PA and be more adherent with the accelerometer. Additionally, we recognize that correlations have numerous assumptions that may influence the interpretation of data. Finally, the DAS-CRP3 measure of disease activity may not be a complete reflection of current disease state at the time of the accelerometry trial and may have attenuated the reported correlations due to the time difference between sampling of biomarkers (conducted at annual registry visit) and accelerometer intake (an average of 4 months).

In this sample, adults with RA did not meet the ACSM requirements for PA despite relatively well-controlled RA disease. The relative ease of completing the NHSPAQ and short survey length, along with modest correlations with accelerometry data, suggests the NHSPAQ could potentially be useful in large epidemiological studies of PA in RA, though further study with larger samples is needed.

## Conclusions

Patients with RA exhibit low physical activity levels. General fitness performance measures were moderately correlated with self-reported physical activity levels and with accelerometer data. A moderate significant correlation existed between NHSPAQ derived METs and accelerometry METs. These preliminary data suggest the NHSPAQ may be useful to describe physical activity levels in this group of patients.

## Abbreviations

ACSM: American College of Sports Medicine
DAS-CRP3: Disease Activity Score with C-Reactive Protein
IQR: Inter Quartile Range
MDHAQ: Multidimensional Health Assessment Questionnaire
METs: Metabolic equivalents of energy expenditure
MHI-5: Mental Health Status Index
NHSPAQ: The Nurses’ Health Study physical activity questionnaire
PAQ: Physical activity questionnaires
RA: Rheumatoid arthritis
SD: Standard deviation
